# Cholesterol lowering therapies and achievement of targets for primary and secondary cardiovascular prevention in type 2 diabetes: unmet needs in a large population of outpatients at specialist clinics

**DOI:** 10.1186/s12933-020-01164-8

**Published:** 2020-11-10

**Authors:** Mario Luca Morieri, Angelo Avogaro, Gian Paolo Fadini, the DARWIN-T2D Network of the Italian Diabetes Society

**Affiliations:** Department of Medicine, University of Padova, Via Giustiniani 2, Padua, 35128 Italy

**Keywords:** Cardiovascular prevention, Diabetes, LDL cholesterol targets, Risk reduction, Numbers needed to treat, PCSK9 inhibitors, Real-world studies, Guidelines

## Abstract

**Background:**

The well-established benefit of Low-Dense-Lipoprotein-cholesterol (LDL-c) lowering treatments (LLTs) has led clinical guidelines to lower the cardiovascular prevention targets. Despite this, there is a surprising scarcity of real-world studies (RWS) evaluating whether recommendations are applied in the routine clinical management of patients with type 2 diabetes (T2D). We therefore evaluated, in a large RWS, the pattern of LLTs use and the achievement of LDL-c targets in patients with T2D in Italian diabetes specialist clinics.

**Methods:**

We collected data from 46 diabetes outpatient clinics (following 281,381 subjects), including 104,726 T2D patients, for whom use of LLTs between 2015 and 2016 was ascertained. We used the 2016 and 2019 European Atherosclerosis Society and European Society of Cardiology (EAS-ESC) guidelines to define cardiovascular risk categories, LDL-c targets, and the expected LDL-c reduction and cardiovascular benefit achievable with LLT intensification.

**Results:**

63,861 patients (61.0%) were on statin therapy, 9.2% of whom were also on ezetimibe. Almost all subjects were at high (29.3%) or very high (70.4%) cardiovascular risk, including 17% being in secondary prevention. Among very high-risk patients, 35% were not on statin despite half of them had LDL-c > 2.6 mmol/l, and only 15% of those on statins had LDL-c < 1.4 mmol/l. 83% of subjects in secondary prevention were on a statin, but half of them had LDL-c > 1.8 mmol/l. Overall, 35% and 14% of subjects achieved the LDL-c targets as suggested by 2016 and 2019 EAS-ESC Guidelines, respectively. Based on anticipated response to treatment, we estimated that 38% of the entire population would require high-intensity-statin (HI-statin), 27% a combination of HI-statin plus ezetimibe, and 27% the addition of proprotein-convertase-subtilisin/kexin-9 (PCSK9) inhibitors. These LLT intensifications would reduce the incidence of cardiovascular events by 32%, from 23.511 to 16.022 events per 100.000 patients/10-years (incidence-rate-ratio 0.68; 95% C.I 0.67–0.70, p < 0.001).

**Conclusions:**

Despite the increase in use of LLT in T2D over the last decades, a large proportion of subjects with T2D did not achieve their LDL-c targets. Given the very high cardiovascular risk of these patients, improving LLT is expected to have a dramatic impact on cardiovascular event prevention.

## Introduction

As patients with type 2 diabetes (T2D) have a ~ twofold increased risk of CVD compared to those without T2D [1], timely and adequate control of CVD risk factors is of primary relevance in these individuals [2, 3]. Lipid-lowering therapy (LLT), aimed to reduce LDL-cholesterol levels is an essential cornerstone of preventive strategies both in the general population and in those with T2D [3, 4]. Indeed, LDL-cholesterol is one of the main causal and modifiable factors associated with CVD events [5]. The extensively confirmed role of LDL-cholesterol as a causal factors for atherogenesis [6] led scientific societies to reduce progressively the levels of LDL-c targets for cardiovascular prevention [2, 3]. This approach is justified by the very-well documented dose-dependent log-linear relationship between the reduction of LDL-c levels and the relative risk reduction (RRR) of developing future CVD events [6, 7]. A relationship that has been confirmed both in patients with and in those without T2D [7, 8]. Moreover, in patients with T2D, who have a higher CVD risk compared to patients without T2D, the clinical benefit derived from the same LDL-c reduction is higher than that achievable in subjects without T2D benefit (i.e. the same RRR is translated in an higher absolute risk reduction [ARR] given the higher CVD risk). Despite this, in routine clinical practice, LDL-c levels frequently remain far from the targets recommended by scientific societies. In the Italian registry DIARIO [9], only 20.4% of 10,000 subjects with T2D, evaluated at outpatient specialist diabetes clinics in 2002, were treated with a statin. As of today, there is a lack of similar surveys evaluating the modern use of LLT and the achievement of LDL-c levels in patients with T2D in European countries and in Italy.

Recently, the Italian Society of Diabetes has promoted the multicentre observational study DARWIN-T2D (DAta for Real World evIdeNce in Type 2 Diabetes), allowing the evaluation of routinely accumulated data of up to ~ 281,000 subjects with T2D attending diabetes specialist clinics in Italy [10]. Leveraging the resources of DARWIN-T2D, we herein analysed the current use of LLT, the LDL-c levels and achievement of recommended LDL-c targets according to presence or absence of concomitant CVD risk factors and the CVD risk categories suggested by European Society of Cardiology (ESC) and the European Atherosclerosis Society (EAS) Guidelines. Eventually, the aim of this study was to evaluate whether implementation of LLT would allow the achievement of recommended LDL-c targets, and to estimate the clinical benefit, in terms of reduction of future cardiovascular events, that would arise from such interventions.

## Methods

### Population

The DARWIN-T2D study collected data from 46 diabetes outpatient clinics in Italy, for a total of 281,381 patients [10]. In this retrospective study, we extracted information from patients aged 18–80 years, with T2D since at least 1 year (as recorded in the chart), and for whom use (or not) of LLT was known between 2015 and 2016. No information were available on treatment dosage, such that high and low-medium intensity statin treatment were defined according to statin molecules as follow: rosuvastatin and atorvastatin (high-intensity, HI-statin) and simvastatin, pravastatin, lovastatin, fluvastatin (as moderate-intensity MI-statin). Information on therapy prescription were available, while data on adherence to treatment were not (e.g. whether the patients refilled treatments from pharmacy, or whether they actually took the drugs). At the time of data collection, poprotein-convertase-subtilisin/kexin-9 inhibitors (PCSK9-inhibitors) were not available in the market Italy, and therefore no patients were treated with PCSK9i. The study protocol conforms to the ethical guidelines of the 1975 Declaration of Helsinki and was approved by the local ethical committees at participating centers. The study used anonymous data and, based on National and International regulations, a waiver was applied to the requirement for patients’ informed consent.

Further details on the collected information on demographics, anthropometrics, laboratory exams and comorbidities (including ICD9 code used) are described in supplemental methods. Briefly, presence of coronary heart disease (CHD) was defined as a history of angina or myocardial infarction or coronary revascularization. Microangiopathy was defined as the presence of nephropathy, retinopathy/maculopathy or neuropathy, as defined in previous reports [10, 11]. Previous cardiovascular disease (CVD) was defined as a history of stroke or myocardial infarction or any site revascularization. Macroangiopathy was defined as history of prior CVD events, or cerebral, coronary or peripheral atherosclerosis, even if asymptomatic.

### Definition of risk categories, LDL-targets and required treatment improvement

Cardiovascular risk category were defined accordingly to the 2019 ESC-EAS Guidelines on Dyslipidemia as very high (VH) risk, high (H) risk, and medium risk [12]. The same guidelines were used to define LDL-cholesterol targets in each EAS-ESC CVD risk categories (i.e. 50% reduction from untreated levels and LDL-c < 1.4 mmol/l in VH-risk group and < 1.8 mmol/l in H-risk group). Results were also put in the context of previous targets as specified in the 2016 EAS-ESC Guidelines on dyslipidemia that were effective at that time these data were collected [13]. The expected reduction with specific lipid-lowering treatments, as defined by EAS-ESC guidelines was considered, from a treatment naïve LDL-c level as follow: 30% reduction after MI-statin, 50% after HI-statin, 65% after HI-statin + Ezetimibe, and 85% after HI-statin + Ezetimibe + PCSK9i [12] (more details on these average LDL-c reductions expected by use of different treatments and combinations, as reported by EAS-ESC dyslipidemia guidelines, are summarized in Additional file 1: Table S1). For subjects who were already on LLT at the time of the survey, the theoretical naïve LDL-c levels was evaluated backwards using the inverse of the  % expected reduction with the current treatments, i.e. naïve LDL-c = actual LDL-c/(1-expected reduction with current treatments). Therefore, the achievable LDL-c or  % reduction with implementation of treatments were evaluated as reported in Additional file 1: Table S1. For each subject, we then evaluated which treatments would have been required to reach the LDL-c reduction as suggested by current guidelines. This evaluation was based on the distance to target (DTT) from current LDL-c levels and the LDL-target. The estimated 10-years risk of fatal and non-fatal CVD events (including fatal and non-fatal myocardial infarction, stroke and cardiovascular death) was evaluated according to the Progetto Cuore CVD risk score [14], which is specific for the Italian population.

### Statistical analyses

Continuous variables are presented as mean and standard deviation if normally distribution or as median and interquartile range, whereas categorical variables are presented as percentages. The comparison of characteristics between two groups was performed using Student’s t test for continuous variables and Chi square test for categorical variables. Non-normal variables were log-transformed before analysis. To evaluate the relationship between age and sex with the achievement of LDL-cholesterol targets, we used Poisson regression models with a robust error variance including age, sex, concomitant LLTs, and presence of micro- and macro-angiopathy as covariates. The baseline absolute risk of CVD events over 10-years in each group of interest was defined by the median value of the risk estimated with the “Progetto Cuore” CVD risk score. This value was used to estimate the expected number of subjects that would experience a CVD event over 10 years. In each group, starting from the current LDL-c levels, we estimated the absolute LDL-c reduction (LDLred) that would be achieved following the suggested LLT changes required to reach the LDL-c targets. We then estimated the corresponding RRR, according to the known dose-dependent log-linear relationship between LDL-c reduction and of CVD risk. In the short time period (i.e. 5 years), this is known to be an expected 22% RRR for each 38.67 mg/dl (1 mmol/l) reduction of LDL-c levels, while in a longer period of time (such as 10 years) this can be expected to be a 28% RRR for each mmol/l reduction of LDL-c levels (i.e. RRR = 1-exp(-0.249 + (number of years of treatment–5) * (−0.0152)) * 100) [6, 12]. In each group, we therefore estimated the RRR to be = 1−exp(LDLred/38.7 * ln(0.72)). The estimated ARR was computed starting from the estimated baseline absolute risk (“Progetto Cuore” risk score) and the 95% C.I. of ARR were estimated as previously described [15]. The number needed to treat (NNT) to prevent one major cardiovascular events over 10 year was estimated as 1/ARR. Moreover, given the absolute CVD risk and the ARR achievable with LDL-c reduction, we estimated the total number of events per 100,000 subjects over 10 years that would be avoided with changes in LLT. We reported the incidence rate ratio (IRR) as the ratio between expected incidence rate of events expected with no changes to current treatment Vs that expected after intensification of treatments. A 2-tail *p* value < 0.05 was considered statistically significant. Statistical analyses were performed using SAS version 9.4 (TS1M4), graphs were produced with GraphPad Prism ver. 8.

## Results

### Patient characteristics

**Fig. 1:**
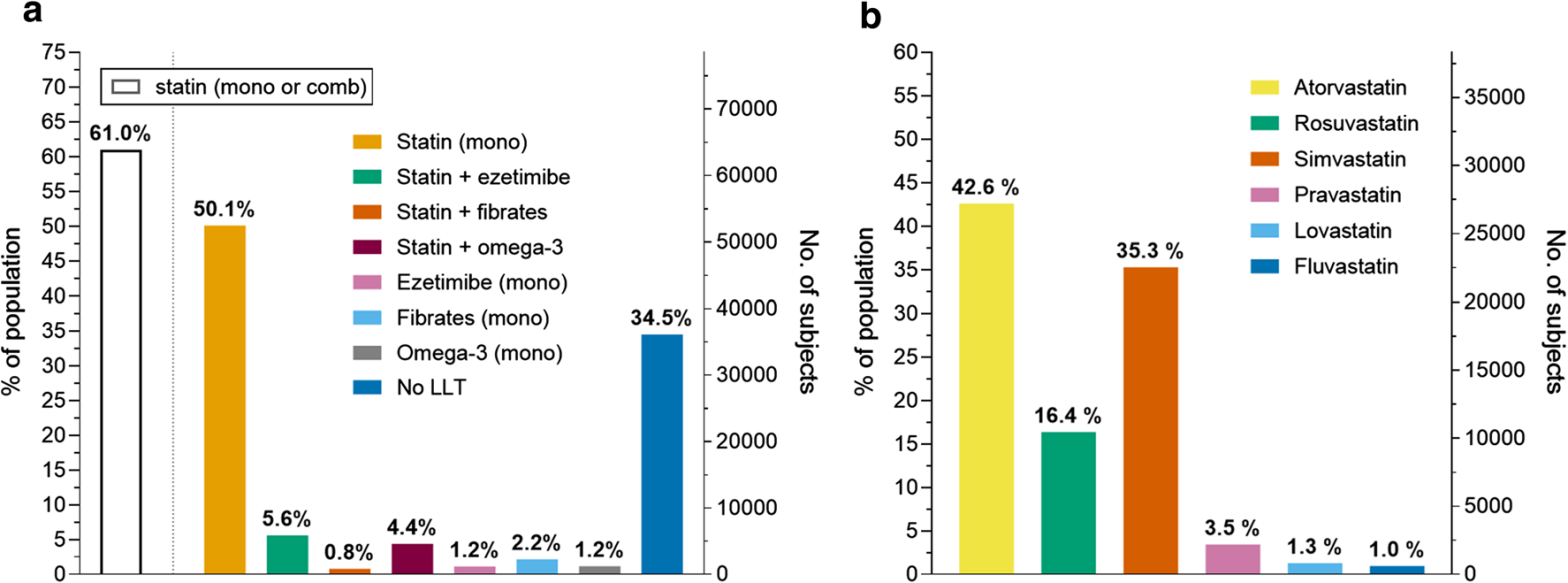
Prevalent use of lipid-lowering treatments (**a**), and of specific statin molecules (**b**)

The study included 104,726 patients with T2D and with available information on the use of LLT (Additional file 1: Figure S1). The mean (SD) age was 70.0 (10.8) years, 56.7% were male, the mean duration of diabetes was 12.4 years, and HbA1c was 7.2%. As shown in Fig. 1, there were 63,861 (61%) patients treated with statin, of whom 59.0% were taking HI-statins (atorvastatin 42.6% and rosuvastatin 16.4%) and 41.0% MI-statins (simvastatin 35.3%, pravastatin 3.5%, lovastatin 1.3% and fluvastatin 1.0%). Among those treated with statins, 5889 (9.2%) subjects were also treated with ezetimibe, 1013 (1.6%) with fibrates and 5702 (8.9%) with omega-3 fatty acid. There were 1217 (1.2%), 2366 (2.3%) and 1235 (1.2%) patients on ezetimibe, fibrate, or omega-3 fatty acids monotherapy, respectively. Overall, 34,5% of the population were not taking any lipid-lowering treatment.

Clinical characteristics of patients according to statin treatment are described in Table 1. As expected, patients taking statin were older, with longer duration of diabetes, and higher prevalence of renal and cardiovascular disease. Moreover, patients on statin were more frequently men and active smokers than those not treated with statins.

**Table 1:** Clinical characteristics of patients divided according to presence or absence of ongoing statin therapy

	On statin		Not on statin		P
	Available %	Value	Available %	Value	
Demographics					
Number		63,861		40,865	
Age, years	100.0	70.3 ± 9.9	100.0	69.6 ± 11.9	< 0.001
Sex male, %	100.0	57.5	100.0	54.9	< 0.001
Diabetes duration, years	100.0	13.1 ± 9.6	100.0	11.5 ± 9.5	< 0.001
Risk factors					
Active smoker, %	74.7	65.2	76.3	59.2	< 0.001
Obesity, %	93.7	41.8%	91.2	39.8%	< 0.001
BMI, kg/m2	93.7	29.3 ± 5.2	91.2	29.6 ± 5.8	< 0.001
SBP, mm Hg	82.1	137.4 ± 18.4	77.3	137.4 ± 18.5	0.843
DBP, mm Hg	82.1	76.8 ± 9.4	77.2	78.0 ± 9.7	< 0.001
Hypertension, %	96.0	89.8	93.6	91.6	< 0.001
FPG, mmol/L	93.3	7.9 ± 2.4	92.0	8.0 ± 2.6	0.045
HbA1c, %	97.6	7.2 ± 1.1	95.9	7.2 ± 1.3	< 0.001
Total cholesterol, mmol/L	90.4	4.2 ± 1.0	83.2	4.6 ± 1.0	< 0.001
HDL cholesterol, mmol/L	88.8	1.3 ± 0.4	80.6	1.3 ± 0.4	0.030
Triglycerides, mmol/L	89.7	1.6 ± 1.0	82.3	1.6 ± 1.1	0.005
LDL cholesterol, mmol/L	87.2	2.2 ± 0.8	78.8	2.7 ± 0.8	< 0.001
Complications					
Kidney Disease	95.5		91.9		< 0.001
CKD III stage, n (%)		28.6		27.2	< 0.001
eGFR, ml/min/1.73 m2	100.0	72.1 ± 22.2	85.5	73.6 ± 23.0	< 0.001
AER, mg/24 h	92.1	39.2 ± 49.4	86.9	43.4 ± 52.5	< 0.001
AER > 30 mg/g		34.5		37.6	< 0.001
Eye disease:	74.4		64.2		< 0.001
Retinopathy, %		17.8		16.5	< 0.001
DME, %		2.9		2.8	0.407
Neuropathy	34.5		33.0		< 0.001
Peripheral, %		20.0		18.4	< 0.001
Autonomic, %		2.5		3.0	0.003
Lower Limbs:	39.2		33.5		< 0.001
Atherosclerosis obliterans, %		20.9		14.7	< 0.001
Revascularization, %		2.3		1.2	< 0.001
CNS Complications:	53.2		40.2		< 0.001
Stroke/TIA, %		5.5		4.8	0.001
Carotid atherosclerosis, %		45.4		36.2	< 0.001
Cardiac complications;	76.7		65.9		< 0.001
IHD, %		16.7		5.6	< 0.001
Revascularization, %		11.7		2.7	< 0.001
Micro-angiopathy, %	98.4	58.6	96.2	58.0	0.070
Macro-angiopathy, %	80.0	46.1	69.0	30.1	< 0.001
Glucose lowering medications	92.4		89.1		
Insulin %		35.3		34.9	0.294
Metformin %		70.6		67.1	< 0.001
Sulfonylureas %		25.1		24.1	< 0.001
DPP-4i %		21.7		18.1	< 0.001
GLP-1RA %		3.7		3.5	0.077
SGLT2i %		3.0		2.6	< 0.001
Other therapies	100.0		100.0		
APT, %		61.1		35.8	< 0.001
Ezetimibe, %		9.2		3.0	< 0.001
Fibrate, %		1.6		5.8	< 0.001
Omega-3, %		8.9		1.6	< 0.001
ACEi/ARB, %		69.7		62.6	< 0.001
CCB, %		26.7		23.2	< 0.001
Beta-blockers, %		34.9		26.5	< 0.001
Diuretics, %		20.9		18.4	< 0.001

Data are expressed as mean ± standard deviation or as percentage where appropriate

*BMI* body mass index, *SBP* systolic blood pressure, *DBP* diastolic blood pressure, *FPG* fasting plasma glucose, *HDL* high-density lipoprotein, eGFR *estimated glomerular filtration rate, CKD* chronic kidney disease, *DME* Diabetic Macular Edema, *TIA* transient ischemic attack, *CVD* cardiovascular disease, *IHD* Ischemic heart disease, *ACEi* angiotensin converting enzyme inhibitors, *ARBs* angiotensin receptor blockers, *CCB* calcium channel blockers, *APT* anti-platelet therapies

### LDL-c levels and achievement of targets for cardiovascular prevention

**Fig. 2:**
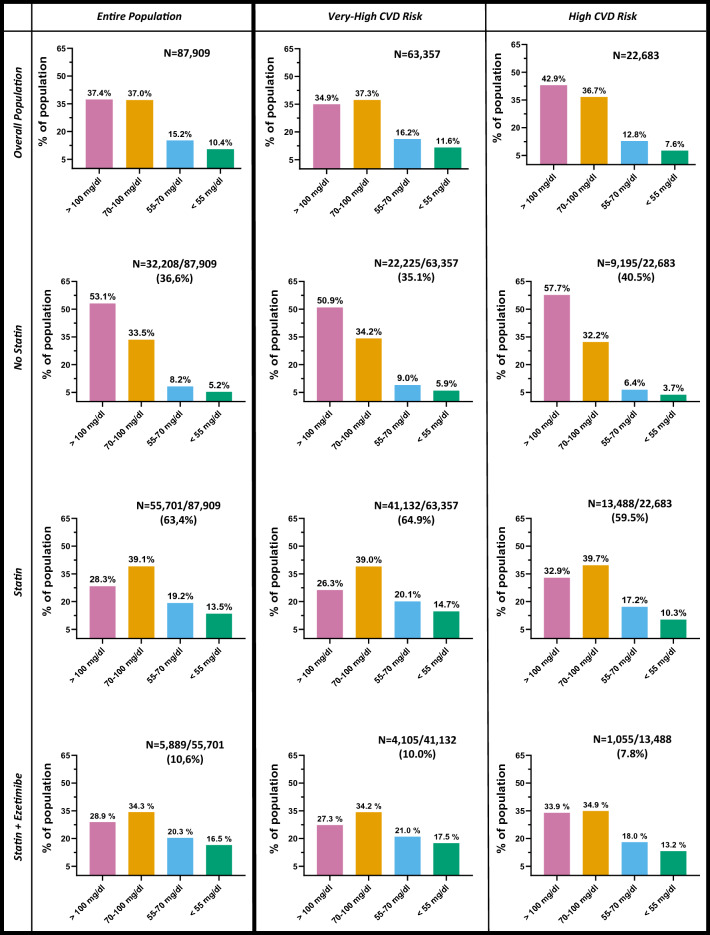
LDL-c levels in the overall population stratified by cholesterol lowering therapy and by ESC/EAS risk categories. For each subgroup of patients, we show numbers and percentages in the various LDL-c target range

LDL-c levels were available in 87,909 (83.4%) of participants (Additional file 1: Figure S1). Among these (Fig. 2, left column), 37.4% of the population had LDL-c levels above 2.6 mmol/l (100 mg/dl). Such proportion raised to 53.1% among those not on statin, while it was 28.3% for those on statin. On the other side, only one out of four patients had LDL-c levels < 1.8 mmol/l (70 mg/dl). Such proportion was 13.4% and 32.7% in the absence or presence of statin treatment, respectively, and 36.8% among those on treatment with statin plus ezetimibe.

The proportion of patients achieving LDL-c targets for cardiovascular prevention was then evaluated according to clinical characteristics. Specifically, patients were stratified according to the EAS/ESC risk categories, presence or absence of macrovascular disease, or previous cardiovascular events, microangiopathy, or ≥ 2 concomitant major cardiovascular risk factors (obesity, smoking, hypertension).

### Achievement of LDL-c targets according to EAS/ESC risk categories

The EAS/ESC CVD risk categories was evaluated in 102,469 subjects (97.8%, Additional file 1: Figure S1). Most patients were at very high CV risk (n = 72,136, 70.4%), about one-third was at high risk (n = 29,993, 29.3%), and less than 1% was at medium risk (n = 340, 0.3%), Clinical characteristics are detailed in Additional file 1: Table S2. As shown in Fig. 2 (middle column), among those at very-high CVD risk, 35% were not treated with statin, despite half of these patients had LDL-c levels above 2.6 mmol/l (100 mg/dl) and less than 15% had LDL-c levels below 1.8 mmol/l (70 mg/dl). Among subjects treated with statins, 26% had LDL-c levels above 2.6 mmol/l (100 mg/dl), while about one-third (35%) had LDL-c < 1.8 mmol/l (70 mg/dl), with only 15% reaching the target of LDL-c < 1.4 mmol/l (55 mg/dl). One out of 10 patients treated with statins were also on concomitant therapy with ezetimibe (n = 4516). However, also among these patients (statin + ezetimibe), only 17.5% achieved the target of LDL-c < 1.4 mmol/l, and only 38.5% achieving the less stringent target of LDL-c < 1.8 mmol/l. The proportion of subjects treated with statins ± ezetimibe was slightly but significantly higher among those with very high CVD risk compared to those with high CVD risk (64.9% *vs* 59.5% and 10.0% *vs* 7.8%, both p < 0.001). We then found that in patients with high or very high CV risk, sex and age were associated with the probability of achieving LDL-cholesterol targets independently from LLT treatments or presence of comorbidities. Indeed, as shown in Additional file 1: Figure S2, among subjects with very high CV risk, a younger age (< 65 years old) and female sex were associated with a 25% and 32% lower probability of achieving LDL-c lower than 55 mg/dl (1.4 mmol/l), respectively (RR 0.75, 95% C.I 0.70–0.80 and 0.68: 95% C.I 0.64–0.71).

A similar use of LLT and achievement of LDL-c targets were found when the population was stratified by presence or absence of micro-angiopathy (at least one among retinopathy, neuropathy or nephropathy) or multiple cardiovascular risk factors (at least 2 among obesity, smoking or hypertension), as shown in Additional file 1: Figures S3 and S4.

### Achievement of LDL-c targets according to macroangiopathy and CVD events

Information on LDL-c levels and clinical or subclinical macroangiopathy were available in 68,107 patients (Additional file 1: Figure S1). Among those with macroangiopathy (n = 27,541; 40.4%), one-fourth was not taking statins despite the vast majority (82.3%) had LDL-c levels > 1.8 mmol/l (70 mg/dl) (Additional file 1: Figure S5). When treated with statins, the proportion of patients with LDL-c < 1.8 mmol/l (70 mg/dl) was twice as much that of untreated subjects (37% *vs* 18%) and raised to 42% among those on statin + ezetimibe treatment. However, also within patients treated with statins ± ezetimibe, less than 20% reached the optimal targets of LDL-c < 1.4 mmol/l (55 mg/dl), with more than 20% of subjects having LDL-c > 2.6 mmol/l (100 mg/dl).

**Fig. 3:**
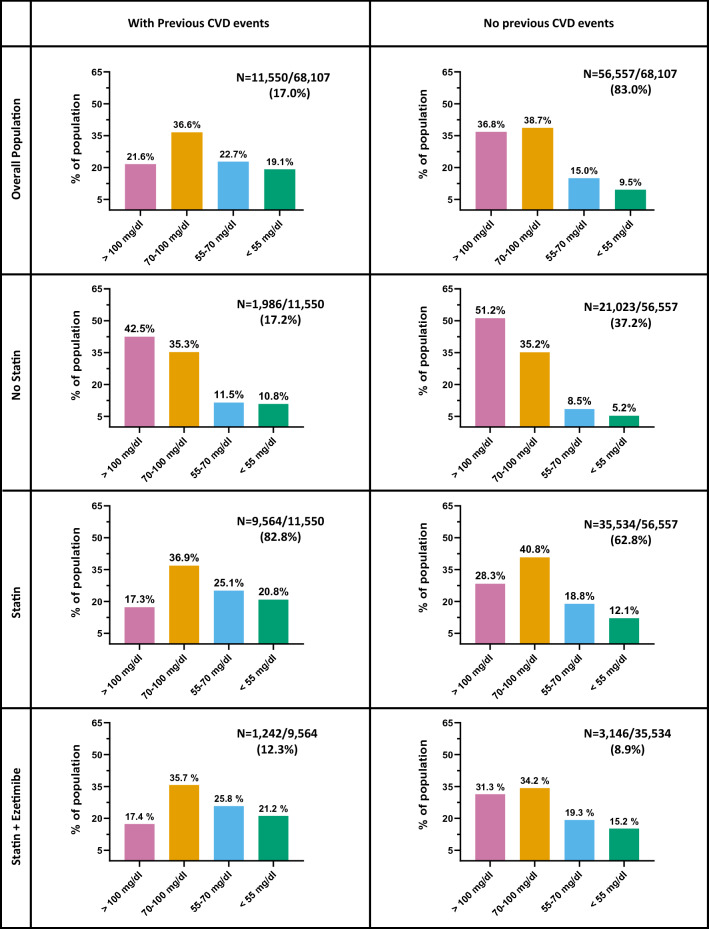
LDL-c levels in the population stratified by therapy and prior major CVD events. For each subgroup of patients, we show numbers and percentages in the various LDL-c target range

As described in Fig. 3, among patients with a previous history of CVD (n = 11,550, 17% of the entire population), 17% where not treated with a statin. However, most of these patients had LDL-c > 1.8 mmol/l (70 mg/dl) and only 10% had LDL-c < 1.4 mmol/l (55 mg/dl). The combined use of statin plus ezetimibe was more common among patients in secondary prevention as compared to those in primary prevention (1241/11,550 [10.8%] vs 3146/56,557 [5.6%], p < 0.001), and more frequently associated with LDL-c levels below 55 mg/dl (21.2% vs 15.2%, p < 0.001). Nonetheless, among patients with prior history of CVD, more than half of subjects treated with statin ± ezetimibe had LDL-c > 1.8 mmol/l (70 mg/dl). In this group of subjects in secondary prevention, female sex and younger age were independently associated with a lower probability of achieving LDL-c < 55 mg/dl (RR 0.68 95% C.I 0.62–0.75, p < 0.001 and RR 0.89; 95% C.I 0.81–0.998, p = 0.046).

### LDL-c targets and intensification of treatments

Altogether, according to CVD risk categories (Table 2), current treatments and LDL-c levels, only 35.9% of the population achieved the LDL-c targets suggested by the 2016 ESC/EAS guidelines (i.e. those available in the years in which these data are referred to). Up to date targets (i.e. those suggested the 2019 Guidelines), were reached at most by 13.9% of the population (if one considers only the absolute LDL-c levels, independently of the recommended required LDL-c reduction).

**Table 2:** Treatments required to achieve the LDL-c targets and the expected clinical benefits

LDL-c group	Current therapy	10 yr CVD risk	Mean LDL-c	2016 GLT	2019 GLT	DTT %	Treatment needed to reach target	LDL-c red	Expected ARR (95% C.I.)	Expected NNT (95% C.I.)
Very high risk										
	No Statin (n = 1319; 1.5%)	25%	1.1	√	(√)	50%	Hi-St	0.6	4.3%	30.0
< 1.4	Mi-St (n = 1268; 1.5%)	28%	1.2	√	(√)	28%	Hi-St	0.3	2.8%	46.3
	Hi-St (n = 4051; 4.7%)	26%	1.1	√	√	–	–	–	–	–
	Mi-St + Eze (n = 574; 0.7%)	23%	1.1	√	√	–	–	–	–	–
	Hi-St + Eze (n = 144; 0.2%)	22%	1.2	√	√	–	–	–	–	–
Sub-Total n = 7356; 8.5%									1.2 (− 0.2 –2.7)	80 (38 –− 638)
	No Statin (n = 1992; 2.3%)	25%	1.6	√		50%	Hi-St	0.8	5.8%	21.7
1.4–1.8	Mi-St (n = 2470; 2.9%)	25%	1.6	(√)		28%	Hi-St	0.5	3.5%	37.0
	Hi-St (n = 4916; 5.7%)	26%	1.6	√		14%	Hi-St + Eze	0.5	3.9%	33.1
	Mi-St + Eze (n = 710; 0.8%)	26%	1.6	√		14%	Hi-St + Eze	0.6	4.5%	28.4
	Hi-St + Eze (n = 153; 0.2%)	26%	1.6	√		14%	Hi-St + Eze + PCSK9i	0.9	6.6%	19.0
Sub-Total n = 10,241; 11.9%									4.3 (3.1–5.4)	24 (19 – 33)
	No Statin (n = 7592; 8.8%)	25%	2.2			50%	Hi-St	1.1	7.6%	16.6
1.8–2.6	Mi-St (n = 5650; 6.5%)	25%	2.2			28%	Hi-St	0.6	4.4%	28.8
	Hi-St (n = 8993; 10.4%)	25%	2.2			35%	Hi-St + Eze + PCSK9i	1.5	9.9%	12.6
	Mi-St + Eze (n = 1150; 1.3%)	24%	2.2			35%	Hi-St + Eze	0.8	5.4%	23.4
	Hi-St + Eze (n = 255; 0.3%)	26%	2.2			36%	Hi-St + Eze + PCSK9i	1.2	8.5%	14.7
Sub-Total n = 23,640; 27.4%									7.6 (6.9–8.3)	13 (12 –15)
	No Statin (n = 11,322; 13.1%)	25%	3.2			57%	Hi-St + Eze	2.1	12.5%	9.7
>2.6	Mi-St (n = 3964; 4.6%)	24%	3.2			57%	Hi-St + Eze + PCSK9i	2.6	13.8%	8.7
	Hi-St (n = 5715; 6.6%)	26%	3.3			58%	Hi-St + Eze + PCSK9i	2.3	13.8%	8.7
	Mi-St + Eze (n = 892; 1.0%)	24%	3.5			60%	Hi-St + Eze + PCSK9i	2.5	13.3%	9.0
	Hi-St + Eze (n = 227; 0.3%)	28%	3.5			60%	Hi-St + Eze + PCSK9i	2.0	13.1%	9.3
Sub-Total n = 22,120; 25.6%									13.1 (12.4 - 13.8)	7.6 (7.2 - 8.1)
Total very-high risk n = 63,357; 73.4%		25%							8.3 (7.8–8.7)	12.1 (11.5–12.8)
High risk										
	No Statin (n = 341; 0.4%)	19%	1.1	√	(√)	50%	Hi-St	0.6	3.1%	32.0
< 1.4	Mi-St (n = 302; 0.4%)	18%	1.2	√	(√)	28%	Hi-St	0.3	1.8%	56.2
	Hi-St (n = 943; 1.1%)	18%	1.2	√	√	–	–	–	–	–
	Mi-St + Eze (n = 109; 0.1%)	15%	1.1	√	√	–	–	–	–	–
	Hi-St + Eze (n = 30; 0.0%)	14%	1.1	√	√	–	–	–	–	–
Sub-Total n = 1725; 2.0%									0.9 −1.6–3.5)	107 (29– −62)
	No Statin (n = 588; 0.7%)	17%	1.6	√	(√)	50%	Hi-St	0.8	4.0%	25.1
1.4–1.8	Mi-St (n = 705; 0.8%)	17%	1.6	√	(√)	28%	Hi-St	0.5	2.3%	42.6
	Hi-St (n = 1421; 1.6%)	20%	1.6	√	√	–	–	–	–	–
	Mi-St + Eze (n = 163; 0.2%)	15%	1.6	√	√	–	–	–	–	–
	Hi-St + Eze (n = 27; 0.0%)	26%	1.6	√	√	–	–	–	–	–
Sub-Total n = 2904; 3.4%									1.4 (− 0.6 –3.3)	73 (30 – −173)
	No Statin (n = 2964; 3.4%)	19%	2.3	√		50%	Hi-St	1.1	5.7%	17.5
1.8–2.6	Mi-St (n = 2045; 2.4%)	18%	2.2	(√)		28%	Hi-St	0.6	3.3%	30.4
	Hi-St (n = 2936; 3.4%)	19%	2.2	√		18%	Hi-St + Eze	0.7	3.7%	27.4
	Mi-St + Eze (n = 302; 0.4%)	17%	2.2	√		18%	Hi-St + Eze	0.8	3.8%	26.7
	Hi-St + Eze (n = 66; 0.1%)	18%	2.2	√		19%	Hi-St + Eze + PCSK9i	1.3	6.0%	16.5
Sub-Total n = 8313; 9.6%									4.3 (3.2–5.4)	23 (18–31)
	No Statin (n = 5302; 6.1%)	19%	3.3			50%	Hi-St	1.6	7.9%	12.7
>2.6	Mi-St (n = 1806; 2.1%)	17%	3.3			45%	Hi-St + Eze	1.6	7.2%	13.9
	Hi-St (n = 2275; 2.6%)	18%	3.3			47%	Hi-St + Eze + PCSK9i	2.3	9.8%	10.2
	Mi-St + Eze (n = 288; 0.3%)	16%	3.4			48%	Hi-St + Eze + PCSK9i	2.5	9.2%	10.9
	Hi-St + Eze (n = 70; 0.1%)	15%	3.4			48%	Hi-St + Eze + PCSK9i	2.0	7.2%	14.0
Sub-Total n = 9741; 11.3%									8.2 (7.2–9.2)	12 (11–14)
Total high risk n = 22,683; 26.3%		18%							5.4 (4.7–6.0)	18.7 (16.6–21.3)
Total n = 86,040; 99.7%		24%		35%	14%				7.5% (7.1–7.9)	13.4 (12.7–4.1)

(√): Achievement of targets according to 2016 or 2019 Guidelines for absolute levels but not for expected  % reduction from untreated levels

*MI-St* Moderate Statin, *Hi-St* High-Intensity Statin, *Eze* Ezetimibe, *PCSK9i* Proportein convertase subtilisin/kexin type 9 inhibitors, *CVD* risk risk score for major cardiovascular (CVD) events over 10 years, *GLT* Guidelines Targets, *DTT* distance to targets from mean LDL-c levels (in  %), *LDL-c red* Expected absolute LDL-c reduction estimated as the product of current LDL-c levels with expected  % reduction with new treatments). Expected ARR: Absolute Risk Reduction (estimated as current absolute CVD risk * RRR, where Relative risk Reduction has been estimated according to the expected absolute LDL-c reduction (i.e. 28% per each mmol/l reduction in LDL-c). NNT: number of subjects needed to be treated to avoid one CVD events over 10 years. 1 mmol/L LDL-c = 38.67 mg/dl

The population was stratified by CVD risk groups, LDL-cholesterol levels (mmol/l) and current treatments

Then, we computed which treatments would be required for each patient to reach the individualized LDL-c percent reduction and absolute targets suggested by current guidelines. First, we found that 8.7% of the population would not require treatment intensification, while around one-third (37.8%) would require the initiation (or shift towards) a HI-statin (including those with apparently absolute LDL-c on target but not the 50% reduction from their estimated untreated LDL-c levels). Second, more than one-fourth of the population (26.9%) would require a combination of HI-statin + ezetimibe, and another one-fourth (26.6%) the further add-on of PCSK9i to reach the recommended LDL-c levels (Table 2).

**Fig. 4:**
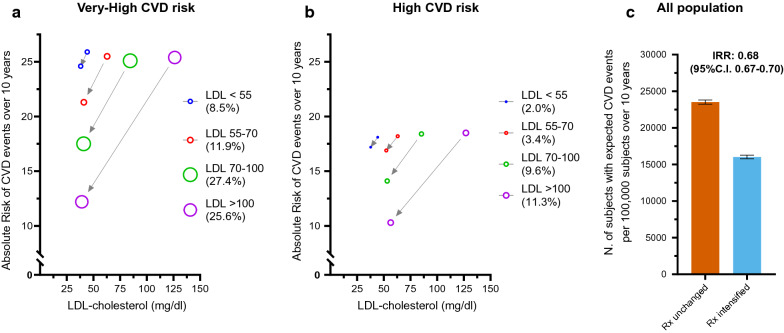
Expected benefit of recommended LDL-c reduction. Panel **a** and **b** shows the absolute risk reduction achievable with treatments aimed to reach LDL-c targets in each group stratified by starting LDL-c levels (number in brackets are the proportion of subjects each group over the entire population). Panel **c** Expected number of subjects experiencing CVD events over 10 years (/100,000 subjects) in case of unchanged or changed cholesterol-lowering treatments

Eventually, we evaluated the expected clinical benefit that would derive from such intensifications of LLT. As shown in Table 2, the clinical benefit, expressed as ARR or NNT to prevent one CVD events over 10 years, was progressively greater among those with higher CVD risk and higher LDL-c levels. This has been also depicted graphically in Fig. 4 panel a and b, showing how the absolute risk would change after intensification of LLT. For instance, intensifying therapy in subjects with very-high risk with LDL-c > 2.6 mmol/l (> 25% of the entire population) would lead to an ARR of 13.1% (95% C.I 12.4–13.8%) and therefore a NNT to avoid 1 CVD events over ten year as low as 7.6 (95% C.I 7.2–8.1). Also among subjects with high risk, the LLT intensification would produce a reduction of 5.4% in the absolute risk (95% C.I 4.7–6.0, p < 0.001). In the entire population (Fig. 4, panel c), LLT intensification would reduce the incidence of CVD by 32%, from 23,511 events per 100.000 patients/10 years to 16,022 per 100.000 patients/10 year (IRR: 0.68; 95% C.I 0.67–0.70).

## Discussion

This large survey, well representative of the total population of patients with T2D attending specialist diabetic clinics in Italy, conveys three clinically relevant messages. First, despite the increase over time in statin use, the current prescription rate is still below expectations and only in a minority of subjects achieved LDL-c targets. Second, LDL-c targets appear to be achievable with implementation of treatments using the current available drugs in most patients with T2D. Third, such implementation would dramatically reduce the impact of CVD among subjects with diabetes.

Leveraging the data collected from a large study conducted in ~ 281,000 patients with T2D between 2015 and 2016, we found that among ~ 100,000 subjects with data on LLT, 61% were treated with statins (with an additional 1.2% in ezetimibe alone) PCSK9 inhibitors were reimbursed by Italian national health systems since 2017. This proportion is remarkably higher compared to the 21% of subjects treated with a statin that was found in a previous report of a similar survey collected from Italian Diabetes Clinics over 15 years ago (DIARIO Study) [9]. However, one should consider that almost all patients included in this survey (99.7%) were at high or very high CVD risk. Therefore, since the current guidelines suggest that those patients with high or very-high CVD risk should obtain a LDL-c reduction of at least 50% regardless of baseline LDL-c levels, the proportion of subjects treated with HI-statin (perhaps in combination with ezetimibe) should be close to 100%. As opposed to this, most patients not on statin had LDL-c above the LDL-cholesterol targets effective in the 2016 guidelines, and also among those treated with statins, 40% were treated with MI-statins, that usually do not allow the required 50% reduction in LDL-c [12]. The proportion of subjects treated with statin was slightly higher among those with very-high CVD risk (65%) and highest in the subgroup of patients with previous history of CVD (83%) who were 17% of the entire population. A similar proportion was also found in the EUROASPIRE-V survey, where among  ~ 8000 patients with coronary events (including 29% with diabetes) from 27 European countries, the frequency of statin use was 80% [16]. Compared to previous reports in T2D, also in this group of patients the prescription of statin was increased (83% *vs* 60–66%) [9], but yet almost 1 out of 5 patients with previous CVD was not receiving any LLT.

Altogether, the LDL-c targets suggested by the 2019 EAS/EAS and EASD Guidelines for dyslipidaemia and cardiovascular prevention [2, 12], were achieved in 14% of the population. Even considering the less stringent targets suggested by previous guidelines (i.e. EAS/ESC 2016 Guidelines for Dyslipidaemia [13]), that were in use at the time this data were collected, they were achieved only by about 35% of patients. Moreover, we found that younger age and female sex were consistently associated with lower probability of achieving LDL-c targets, also within group of subjects with very-high CVD risk. Given the equivalent efficacy of LLT regardless of age or sex [7, 17], one of the possible explanation for this finding might reside in therapeutic inertia (TI), i.e. the failure to intensify treatments when treatment targets are not met [18, 19]. Indeed, TI in the management of dyslipidaemia is higher in subjects with younger age and in females [20]. Despite its multifactorial causes [19], one important point might be the underestimation of CVD risk in these patients [21, 22], who would rather derive important benefits from adequate LLT.

Our data also allow estimating the changes in LLT that would be required to reach the recommended LDL-c targets in each sub-group of patients according to CVD risk, current LDL-c levels, and ongoing LLT. These analysis shows that, on average, according to the expected response in LDL-c reduction, all patients would be able to reach the recommended targets by use of the current available therapeutic armamentarium. Specifically, more than one-third of patients would require initiation or shifting to HI-statin, one-fourth the combination of HI-statin + ezetimibe, and one-fourth (26.7%) also the addition of a PCSK9-inhibitor.

Importantly, we estimated the clinical benefits that could result from implementation of current guidelines in a large population of subjects with T2D. After intensifying LLT, the expected reduction of LDL-c levels would reduce the number of subjects experiencing a CVD events over 10 years by one-third (IRR 0.68; 95% C.I 0.67–0.70). Indeed, despite the limits of the observational cross-sectional design of this study, we used the well-established linear relationship between LDL-c reduction and reduction in CV risk (as described also in EAS/ESC Guidelines on dyslipidaemia [3]) to estimate the expected benefit from LLT improvement. In absolute numbers, this means that if the 100,000 subjects included in this survey followed the recommended LLT, over the next 10 years, more than 7000 patients would avoid a major cardiovascular event (i.e. an ARR of 7.5%; 95% C.I 7.1–7.9%). Translated to the total population of patients with diabetes attending specialist clinics in Italy (approximately 1.9 millions [23]), this approach might protect 130.000 individuals who would otherwise experience a major cardiovascular events over the next 10 years. A benefit that would be most remarkable among subjects with very-high CVD risk and LDL-c above 2.5 mmol/l (100 mg/dl, i.e. ¼ of the entire population), where only 8 patients are needed to be treated with HI-statin + ezetimibe + PCSK9-inhibitor to avoid one major CVD event over 10 years (NNT 7.6, 95% C.I 8.6–9.9).

Beyond the benefit of treating LDL-c, patients with T2D require a comprehensive approach for cardiovascular prevention aiming to adequate control other modifiable risk factors (e.g. glycemic and blood pressure levels, body weight and smoking habits), early identification of diabetic complications, and using cardio-protective glucose lowering medications such as sodium/glucose cotransporter 2-inhibitors and GLP-1 receptor agonists [18, 24]. Is therefore possible to speculate that an ideal control of all these factors would reduce the overall cardiovascular risk in this population and therefore would reduce the expected clinical benefit (i.e. ARR) from LLT. Indeed, given the lack of follow-up data, our estimates of the ARR derivable from intensification of LLT in the population included in this study might be overestimated in case CV risk can be reduced by other means. Nonetheless, given the well-established causal relationship between LDL-c and CVD, the relative risk reduction derived from improvement of LLT, would not be affected by changes in the background CV risk [3, 6].

Our study should be put in the context of some aspects that are clinically relevant in the management of LLT. First, the lack of data on statin dosage has limited our possibilities to provide more detailed information on MI vs HI statin treatment. Similarly, the lack of information on treatment adherence is important because, before discussing changes in LLT, is it essential to address patient’s compliance to current prescriptions, in particular for chronic therapies with a poor long-term persistence, such as statins [25]. The suggested treatment might therefore be different in case of low adherence, or statin under-dosage (e.g. atorvastatin 10–20 mg or rosuvastatin 5–10 mg that are not considered high-intensity). However, these limitations have no influence on current data on achievement of LDL-c levels. Second, muscle-related symptoms, that were not available in our dataset, are considered one of the main reasons for poor adherence [26]. Yet, careful evaluation of these symptoms can often identify situations unrelated to statins, which are provided with a good safety profile even in populations at higher risk of adverse events, such as the elderly [26–28]. However, in patients with confirmed intolerance to statins, alternative effective strategies should be sought (e.g. ezetimibe and PCSK9-inhibitors), knowing that the CVD benefit of LDL-c reduction has been confirmed also with these treatments [6, 29, 30]. Another aspect that should be considered for clinical interpretation is the differences between treatment suggested by guidelines and eligibility for reimbursement by national health systems or insurances. For instance, according to current Italian criteria for reimbursement, only patients with diabetes and high or very high CVD risk and LDL-c levels > 100 mg/dl could have PCSK9i reimbursed. This implies that, among the 26.6% of subjects that might require PCSK9i to reach their LDL-c targets, only 58% would have PCSK9i prescription reimbursed. Finally, though we aimed to analyse of LDL-c targets, there is growing evidence that lipid lowering treatments aimed to reduce TG-rich lipoproteins might provide additional CVD benefit [31], particularly in specific selected subgroups of patients with T2D [32, 33]. Therefore, additional studies are required to address the benefit of treating subjects with elevated non-HDL-cholesterol levels, beyond achievement of LDL-c targets. In this category of patients, PCSK9i have been recently found to reduce not only fasting non-HDL-cholesterol levels but also post-prandial concentration of Apo-B containing lipoproteins [34, 35], which might exert cardiovascular benefits beyond that derived from LDL-c reduction [36, 37].

## Conclusion

In conclusion, patients with T2D attending specialist clinics in Italy were almost entirely at high or very high CVD risk. Despite the increase over time in the prescription of LLT to reduce LDL-cholesterol, only few patients achieved the targets for CVD prevention as suggested by guidelines. The therapeutic armamentarium available to date is expected to allow reaching those targets, and such implementation would significantly decrease the CVD burden in this population.

## Supplementary information

**Additional file 1**. Figures and Tables.

## Data Availability

Data will be available from the corresponding authors at a reasonable request.

## Abbreviations

ARR: Absolute risk Reduction
CHD: Coronary heart disease
CI: Confidence Interval
CVD: Cardiovascular disease
EAS: European Atherosclerosis Society
ESC: European Society of Cardiology
HI-statin: High-intensity-statin
LDL-c: Low dense lipoprotein cholesterol
LLTs: LDL-c lowering treatments
MI-statin: Moderate-intensity-statin
NNT: Number of subjects needed to be treated
PCSK9: Proprotein convertase subtilisin/kexin type 9
RRR: Relative risk reduction
RWS: Real-world studies
TI: Therapeutic inertia
T2D: Type 2 diabetes
