# Supplementary material for: Cholesterol lowering therapies and achievement of targets for primary and secondary cardiovascular prevention in type 2 diabetes: unmet needs in a large population of outpatients at specialist clinics
Source: Cardiovasc Diabetol. 2020 Nov 10;19:190. doi: 10.1186/s12933-020-01164-8 (PMC7653689; doi:10.1186/s12933-020-01164-8)
Supplement: Supplementary file 1 — Additional file 1. Figures and Tables. [file 12933_2020_1164_MOESM1_ESM.docx]

“Cholesterol lowering therapies and achievement of targets for primary and secondary cardiovascular prevention in type 2 diabetes. Unmet needs in a large population of outpatients at specialist clinics”

Mario Luca Morieri et al.

**Data supplement**

Supplement Methods:

The following information were also collected in the he DARWIN-T2D study: demographics, anthropometrics, laboratory exams (fasting plasma glucose, HbA1c, total cholesterol, HDL cholesterol, calculated LDL cholesterol, triglycerides, liver enzymes, serum creatinine for the calculation of eGFR using the CKD-EPI equation, urinary albumin excretion rate (UAER) in mg/g of urinary creatinine or equivalent). Chronic Kidney Disease (CKD) was defined by eGFR <60 ml/min/1.73 m^2^. Cerebral and peripheral atherosclerosis was defined as the presence of narrowing plaques of the carotid or leg arteries, respectively; ICD-9 codes were used to define a history of ischemic stroke / transient ischemic attack (TIA) (433-436), myocardial infarction (410-414), heart failure (428); left ventricular hypertrophy, carotid, peripheral or coronary revascularization were coded separately; coronary heart disease (CHD) was defined as a past history of angina or myocardial infarction or coronary revascularization. Microangiopathy was defined as the presence of nephropathy, retinopathy/maculopathy or neuropathy. Previous cardiovascular disease (CVD) was defined as a history of stroke or myocardial infarction or any site revascularization. Macroangiopathy was defined as previous CVD, or cerebral, coronary or peripheral atherosclerosis, even if asymptomatic.

**Table S1. Expected reduction in LDL-c following changes in lipid-lowering treatments.** Note: the percent reduction from the current levels have been derived from information described in 2019 ESC/EAS Guidelines for the management of dyslipidaemias: lipid modification to reduce cardiovascular risk ([1](#_ENREF_1)). MI-Statin: Moderate intensity Statin; HI-Statin: High Intensity Statin

|  | | **New treatments** | | |
| --- | --- | --- | --- | --- |
|  |  | HI-Statin | HI-Statin + Ezetimibe | HI Statin + Ezetimibe + PCSK9i |
| **Current Treatment** | **No Treatment** | -50% | -65 % | -85% |
|  | **MI Statin** | -28% | -50% | -79% |
|  | **MI Statin + Ezetimibe** |  | -36% | -73% |
|  | **HI Statin** |  | -31% | -70 % |
|  | **HI Statin +Ezetimibe** |  |  | -57% |

1. Mach F, Baigent C, Catapano AL, Koskinas KC, Casula M, Badimon L, et al. 2019 ESC/EAS Guidelines for the management of dyslipidaemias: lipid modification to reduce cardiovascular risk. Eur Heart J. 2019 Aug 31. PubMed PMID: 31504418.

**Table S2. Characteristics in patients stratified by ESC/EAS CVD risk categories**. BMI, body mass index. SBP, systolic blood pressure. DBP, diastolic blood pressure. FPG, fasting plasma glucose. HDL, high-density lipoprotein. eGFR, estimated glomerular filtration rate. CKD, chronic kidney disease. DME, Diabetic Macular Edema. TIA, transient ischemic attack. CVD, cardiovascular disease. IHD, Ischemic heart disease ACEi, angiotensin converting enzyme inhibitors. ARBs, angiotensin receptor blockers. CCB, calcium channel blockers. APT, anti-platelet therapies.

|  | **Very high risk** | | **High risk** | | **Moderate risk** | |
| --- | --- | --- | --- | --- | --- | --- |
|  | **Avail (%)** | **Value** | **Avail (%)** | **Value** | **Avail (%)** | **Value** |
| **Demographics** |  |  |  |  |  |  |
| **Number** |  | 72,136 |  | 29,993 |  | 340 |
| **Age, years** | 100.0 | 71.3±10.4 | 100.0 | 67.2±10.9 | 100.0 | 42.7±6.2 |
| **Sex male, n (%)** | 100.0 | 57.9 | 100.0 | 53.6 | 100.0 | 57.8 |
| **Risk factors** |  |  |  |  |  |  |
| **Active smoker, %** | 75.7 | 31.7 | 75.7 | 27.3 | 66.9 | 18.4 |
| **Diabetes Duration, years** | 100.0 | 13.7±9.9 | 100.0 | 10.0±8.4 | 100.0 | 3.6±2.8 |
| **BMI, kg/m2** | 92.9 | 29.5±5.4 | 92.5 | 29.7±5.6 | 93.1 | 25.5±2.8 |
| **SBP, mm Hg** | 80.7 | 137.7±18.5 | 80.0 | 137.6±18.2 | 78.6 | 120.0±12.0 |
| **DBP, mm Hg** | 80.6 | 76.9±9.6 | 80.0 | 78.5±9.4 | 78.5 | 75.3±7.7 |
| **FPG, mg/dl** | 94.0 | 143.7±45.0 | 90.4 | 141.6±44.1 | 91.8 | 136.4±42.9 |
| **HbA1c, %** | 98.3 | 7.2±1.2 | 94.2 | 7.1±1.2 | 94.5 | 7.0±1.5 |
| **Total cholesterol, mg/dl** | 91.0 | 167.3±39.0 | 80.4 | 174.6±38.9 | 84.9 | 182.9±43.4 |
| **HDL cholesterol, mg/dl** | 89.2 | 48.7±14.4 | 77.8 | 50.0±14.0 | 82.7 | 47.2±14.6 |
| **Triglycerides, mg/dl** | 90.3 | 139.8±82.6 | 79.4 | 137.9±106.6 | 84.7 | 159.8±127.4 |
| **LDL cholesterol, mg/dl** | 87.8 | 90.9±32.8 | 75.6 | 97.3±32.8 | 79.5 | 105.4±35.7 |
| **Complications** |  |  |  |  |  |  |
| **Kidney Disease:** | 97.2 |  | 87.5 |  | 89.5 |  |
| **CKD III stage, n (%)** |  | 39.5 |  | 0.0 |  | 0.0 |
| **eGFR, ml/min/1.73 m2** | 100.0 | 67.7±23.5 | 100.0 | 84.9±13.5 | 100.0 | 103.1±15.6 |
| **AER, mg/24h** | 93.6 | 49.3±57.6 | 82.4 | 19.2±4.7 | 87.4 | 46.5±48.9 |
| **AER >30 mg/g** |  | 49.8 |  | 0.0 |  | 0.0 |
| **Eye disease:** | 77.6 |  | 55.0 |  | 48.8 |  |
| **Retinopathy, %** |  | 22.8 |  | 0.0 |  | 0.0 |
| **DME, %** |  | 3.7 |  | 0.0 |  | 0.0 |
| **Neuropathy:** | 39.6 |  | 21.7 |  | 18.5 |  |
| **Peripheral, %** |  | 24.1 |  | 0.0 |  | 0.0 |
| **Autonomic, %** |  | 3.4 |  | 0.0 |  | 0.0 |
| **Lower Limbs:** | 43.1 |  | 24.2 |  | 14.8 |  |
| **Atherosclerosis obliterans, %** |  | 23.3 |  | 0.0 |  | 0.0 |
| **Revascularization, %** |  | 2.4 |  | 0.0 |  | 0.0 |
| **CNS Complications:** | 56.7 |  | 29.6 |  | 22.1 |  |
| **Stroke/ TIA, %** |  | 6.5 |  | 0.0 |  | 0.0 |
| **Carotid Atherosclerosis, %** |  | 52.3 |  | 0.0 |  | 0.0 |
| **CVD Complications;** | 79.5 |  | 57.9 |  | 42.5 |  |
| **IHD, %** |  | 16.9 |  | 0.0 |  | 0.0 |
| **Revascularization, %** |  | 11.2 |  | 0.0 |  | 0.0 |
| **Micro-angiopathy, %** | 99.7 | 83.0 |  | 0.0 |  | 0.0 |
| **Macro-angiopathy, %** | 83.0 | 53.5 |  | 0.0 |  | 0.0 |
| **Glucose Lowering Medications:** | 92.5 |  | 89.1 |  | 84.9 |  |
| **Insulin %** |  | 39.5 |  | 25.5 |  | 31.0 |
| **Metformin %** |  | 64.8 |  | 80.2 |  | 78.2 |
| **Sulfonylureas %** |  | 25.5 |  | 23.4 |  | 12.4 |
| **DPP-4i %** |  | 21.0 |  | 18.7 |  | 18.7 |
| **GLP-1RA %** |  | 3.5 |  | 4.2 |  | 2.9 |
| **SGLT2i %** |  | 2.8 |  | 3.2 |  | 3.1 |
| **Other Therapies:** | 100.0 |  | 100.0 |  | 100.0 |  |
| **APT, %** |  | 57.0 |  | 39.9 |  | 8.7 |
| **Statin, %** |  | 63.7 |  | 55.3 |  | 43.8 |
| **Ezetimibe, %** |  | 7.5 |  | 5.3 |  | 2.1 |
| **Fibrate, %** |  | 3.4 |  | 2.9 |  | 7.4 |
| **Omega-3, %** |  | 8.0 |  | 5.1 |  | 6.8 |
| **ACEi/ARB, %** |  | 69.8 |  | 66.5 |  | 0.0 |
| **CCB, %** |  | 27.1 |  | 23.5 |  | 0.0 |
| **Beta-blockers, %** |  | 34.1 |  | 28.6 |  | 0.0 |
| **Diuretics, %** |  | 23.6 |  | 12.5 |  | 0.5 |

**Figure S1. Study flowchart and patient disposition.**

**Figure S2. Association between Age and sex with the probability of being at LDL-cholesterol levels < 70 mg/dl (1.8 mmol/l) (for subject at High-CV risk, n=22,683) or LDL<55 mg/dl (1.4 mmol/l) (for subjects at very-high CV risk n=52,604).**


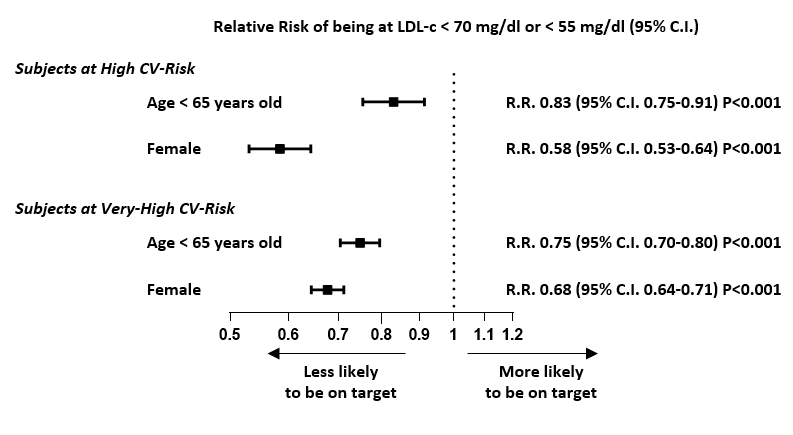


**Figure S3. LDL-c levels in the population stratified by therapy and presence of microangiopathy**. For each subgroup of patients, we show numbers and percentages in the various LDL-c target range.

**Figure S4. LDL-c levels in the population stratified by therapy and risk factors.** For each subgroup of patients, we show numbers and percentages in the various LDL-c target range.

**Figure S5. LDL-c levels in the population stratified by therapy and presence of macroangiopathy.** For each subgroup of patients, we show numbers and percentages in the various LDL-c target range.
